# Dapagliflozin Ameliorates Lipopolysaccharide Related Acute Kidney Injury in Mice with Streptozotocin-induced Diabetes Mellitus

**DOI:** 10.7150/ijms.69031

**Published:** 2022-04-04

**Authors:** Po-Jui Chi, Chung-Jen Lee, Yi-Jen Hsieh, Chia-Wen Lu, Bang-Gee Hsu

**Affiliations:** 1 Division of Nephrology, Department of Medicine, E-DA Hospital, Kaohsiung, Taiwan.; 2 School of medicine, College of medicine, I-Shou University, Kaohsiung, Taiwan.; 3 Institute of Medical Sciences, Tzu Chi University, Hualien, Taiwan.; 4 Department of Nursing, Tzu Chi University of Science and Technology, Hualien, Taiwan.; 5 Division of Nephrology, Hualien Tzu Chi Hospital, Buddhist Tzu Chi Medical Foundation, Hualien, Taiwan.; 6 School of Medicine, Tzu Chi University, Hualien, Taiwan.

**Keywords:** dapagliflozin, diabetes, acute kidney injury, lipopolysaccharide, inflammatory cytokines, reactive oxygen species

## Abstract

Sepsis, which is a serious medical condition induced by infection, has been the most common cause of acute kidney injury (AKI) and is associated with high mortality and morbidity. Sodium-glucose cotransporter 2 (SGLT2) inhibitor is a new oral antidiabetic drug that has greatly improved the cardiovascular and renal outcomes in patients with type 2 diabetes independent of its sugar lowering effect, possibly by attenuation of the inflammatory process. We investigated the effect of the SGLT2 inhibitor dapagliflozin on lipopolysaccharide (LPS)-induced endotoxic shock with AKI in streptozotocin-induced diabetic mice. Endotoxin shock with AKI was induced by intravenous injection of 10 mg/kg LPS in C57BL6 mice with streptozotocin-induced diabetic mellitus with or without dapagliflozin treatment. Observation was done for 48 hours thereafter. In addition, NRK-52E cells incubated with LPS or dapagliflozin were evaluated for the possible mechanism. Treatment with dapagliflozin attenuated LPS-induced endotoxic shock associated AKI and decreased the inflammatory cytokines in diabetic mice. In the *in vitro* study, dapagliflozin decreased the expression of inflammatory cytokines and reactive oxygen species and increased the expressions of AMP-activated protein kinase (AMPK), nuclear factor erythroid-2-related factor, and heme oxygenase 1. These results demonstrated that dapagliflozin can attenuate LPS-induced endotoxic shock associated with AKI; this was possibly mediated by activation of the AMPK pathway.

## Introduction

Sepsis is a complex medical condition that is associated with physiologic, pathologic, and biochemical abnormalities that are induced by infection and usually results in multiple organ failure and septic shock 1. Multiple organ failure, including acute kidney injury (AKI), usually contributes to the high mortality and morbidity in sepsis 1. AKI is the most common complication of sepsis; in fact, 40% to 70% of AKI cases in the intensive care unit were reported to result from sepsis 2. Despite treatment with broad spectrum antibiotics and adequate hydration, the mortality rate of sepsis-associated AKI has remained high and has not improved in the past 50 years.

The mechanisms of sepsis and sepsis-associated AKI are complex. In sepsis, inflammatory mediators derived from pathogens (macromolecular motifs called pathogen associated molecular patterns or PAMPs) and activated immune cells (damage associated with molecular patterns, or DAMPs) can be recognized by pathogen recognition receptors (PRR) on many cells including immune cells, epithelial cell and parenchymal cells. Immune cells are first activated by recognition of pathogens, followed by production of several cytokines, such as tumor necrosis factor α (TNF-α), interleukin 6 (IL-6), and chemokines, enhanced expression of costimulatory receptors essential for efficient T cell activation, production of arachnoid acid metabolites and initiator of extrinsic coagulation cascade, ie. tissue factor 3. An imbalance of cytokines in severe sepsis may cause a severe inflammatory process, intravascular coagulopathy, and thrombus formation 3. The presence of multiple microthrombi would further impair the microcirculation and cause multiple organ failure. Sepsis-associated AKI is related to four pathophysiologic mechanisms: alterations of microcirculatory flow, inflammation, bioenergetic adaptive response to injury, and microparticles 4. Alterations of microcirculation by sepsis increase risk of shunting and develop regional renal hypoperfusion, even in increased renal blood flow, causing AKI. Inflammatory cytokines secreted by immune cells during sepsis will increase endothelial adhesion molecules, lead to leukocytes activation and therefore create a vicious cycle. Bioenergetic adaptive response to injury means a metabolic reprogramming strategy for cells to optimize and reprioritize energy consumption when sepsis, especially for mitochondria-rich organs, such as heart, kidney and liver. However, it will influence the risk of progression to chronic organ dysfunction. Microparticles are intact vesicles released from many cell types including endothelial cells, monocytes, platelets and smooth muscle cells when stimulated by progressive microvascular injury 5, 6. These vesicles contains proteins and lipid from cell membrane with surface antigens 7 and are considered as an effector pools which may exert proinflammatory and prothrombotic events in systemic circulation 8. Microparticles are therefore play a critical role in both initiation and propagation of sepsis and associated AKI. Recent evidence showed that adenosine monophosphate-activated protein kinase (AMPK) was a negative regulator of the bioenergetic reprogramming of immune cells (ie. macrophage and monocyte) and suppressed sepsis development *in vivo* 9. Activation of the AMPK pathway in immune cells interferes the development sepsis and can ameliorate further complications, however, the role of AMPK pathway in renal epithelial cells is undetermined.

Diabetes mellitus (DM), which is characterized by chronic hyperglycemia, is usually complicated with multiple target organ dysfunctions, such as atherosclerosis, nephropathy, retinopathy, and peripheral arterial occlusive disease 10. Several studies showed that DM alone was an independent risk factor of AKI 11. In addition, DM increases the risk of infection and sepsis during hospitalization 12, due to decreased circulation or delayed wound healing 13. The risk of infection in patients with poor controlled type 1 DM is even greater than that with type 2 DM 12. In patients with sepsis or septic shock, DM precipitates the risk of persistent renal failure in patients who develop AKI 14.

Sodium-glucose cotransporter 2 (SGLT2) inhibitor is a new type of oral antidiabetic drug that can inhibit the proximal tubular SGLT2, thereby, reducing the reabsorption of sodium and glucose 15. In addition to its effect on glycemic control, SGLT2 inhibitors have been verified by recent large-scale clinical studies to lower blood pressure, improve glucose toxicity, and induce hemodynamic effects, thereby, improving the cardiovascular and renal outcomes in patients with type 2 DM 16-20. Moreover, a large-scale clinical trial shows the renal protective effect extends to patients with CKD without DM 21. In animal experiments, SGLT2 inhibitors were shown to improve streptozotocin-induced hyperglycemia and attenuate endothelial dysfunction by at least partially reducing oxidative stress during hyperglycemia 22. SGLT2 inhibition results in increased AMPK activity suggesting SGLT2 inhibitors as being useful in treating sepsis along with complications from DM 23-25. In particular, dapagliflozin was demonstrated to slow the renal complications through suppression of renal inflammation in prediabetic rats 26.

Since sepsis and sepsis associated AKI usually cause high mortality and morbidity, especially in patients with type 1 DM. Dapagliflozin seems to exert protective effect beyond sugar control, possibly against oxidative stress and inflammation. To date, there had been no studies on the effect of dapagliflozin on the endotoxic shock- induced AKI in type 1 DM. The aim of our study was to investigate the effects of dapagliflozin on lipopolysaccharide (LPS)-induced endotoxin shock associated AKI in type 1 diabetic mice and to assess the possible molecular mechanism.

## Materials and Methods

### Preparation of animals

Six-week-old male C57BL6 mice weighing 20-25 g were obtained from BioLASCO (Taipei, Taiwan) and maintained in a controlled environment, with temperature at 22°C-25°C and a 12 h/12 h dark-light cycle. Food and water were provided *ad libitum*. The experimental protocols were approved by the Institutional Animal Care and Use Committee of Tzu Chi University of Science and Technology (approval number 2018001).

### Streptozotocin-induced type 1 diabetes mellitus in mice

Mice in DM groups (DM + LPS group and DM + LPS + Dapagliflozin group) were given a single intraperitoneal injection of 150 mg/kg streptozotocin (ab142155, Abcam, USA) dissolved in 0.1-M sodium citrate buffer (pH 4.5) to induce type 1 DM, as previously published 27. Mice were kept in fasting, except for water, four hours before streptozotocin injection. After injection, the mice were closely monitored every 2 hours for 12 hours to observe for fatal hypoglycemia and were provided with normal diet and 10% sucrose water for two days. On experimental day 7, blood obtained from the tail vein of the mice was tested for glucose (Spotchem SP-4430, Arkray, Minneapolis, MN, USA) to confirm the success of diabetes induction. DM was diagnosed if blood sugar over 250 mg/dL for 3 consecutive readings.

### Blood vessel catheterization

On experimental day 8, animals were anesthetized (Matrx VIP 3000, Midmark, Dayton, OH, USA) by isoflurane (Forane, Baxter, Deerfield, IL, USA) inhalation for 15 minutes. During the period of anesthesia, the femoral artery and vein were cannulated with polyethylene-10. The femoral artery cannula was connected to a pressure transducer to record the arterial pressure on a high-resolution laboratory data recorder (E-corder 410, eDAQ, NSW, Australia). After the operation, the animals were placed in a metabolic cage. Endotoxic shock was induced 24 hours later in the conscious state 28, 29.

### Endotoxin shock

Endotoxin shock was induced by intravenous injection of LPS (*Escherichia coli* O111:B4; L2630, Sigma-Aldrich, St. Louis, MO, USA) dissolved in 0.2 mL of normal saline at a dose of 10 mg/kg 28, 29. The animals were continuously observed for 48 hours after LPS administration and sacrificed thereafter.

### Experimental design

In this study, 24 male C57BL6 mice were randomly divided into the Vehicle, DM + LPS, and DM + LPS + Dapagliflozin groups. On experimental day 8, seven-week-old, mice in the Vehicle group (n=8) were intraperitoneally given with 0.1-M sodium citrate buffer and then intravenously administered with 0.2-mL normal saline for 30 minutes, followed by 0.1-mL normal saline for 30 minutes. Mice in the DM + LPS group (n=8) were streptozotocin induced type 1 DM and intravenously administered with 10 mg/kg of LPS in 0.2-mL normal saline, followed by 0.1-mL normal saline for 30 minutes. Mice in the DM + LPS + Dapagliflozin group (n=8) were streptozotocin induced type 1 DM and intravenously administered with 10 mg/kg of LPS in 0.2-mL normal saline for 30 minutes, followed by 10 mg/kg of dapagliflozin (Biorbyt Ltd., Cambridgeshire, United Kingdom) in 0.1-mL normal saline for 30 minutes 25.

### Transcutaneous glomerular filtration rate assessment in conscious mice

At 48 hours after LPS administration, the animals were anesthetized by isoflurane inhalation using a vaporizer (Matrx VIP 3000, Midmark, Dayton, OH, USA) for transcutaneous glomerular filtration rate (GFR) assessment. After removing hair from the mice, the transdermal GFR monitor (MediBeacon GmbH, Mannheim, Germany) was attached. Fluorescein isothiocyanate (FITC)-sinistrin (0.15 mg/g mouse body weight) (MediBeacon GmbH, Mannheim, Germany) was administered by retro-orbital injection. After observation for 1.5 hours, the device was removed. Transcutaneous GFR was calculated using the half-life derived from FITC-sinistrin clearance curves 30.

### Serum biochemistry and inflammatory cytokines assessment

At 48 hours after LPS administration, the mice were sacrificed, and blood samples were collected and centrifuged at 12,000 g for 10 minutes at 4°C to separate the serum. A portion of the serum sample was tested for glucose, blood urea nitrogen (BUN), creatinine, glutamate oxaloacetate transaminase (GOT), and glutamic pyruvic transaminase (GPT) using a biochemistry analyzer (Spotchem SP-4430, Arkray, Minneapolis, MN, USA) 31. The rest of the serum sample was preserved at -80 °C for measurements of TNF-α, IL-1β, and IL-6 by enzyme-linked immunosorbent assay kits (ab 208348, ab197742, ab222503, Abcam, Cambridge, MA, USA) 29.

### Histopathological and immunohistochemical examination

After sacrificing the mice, the kidneys were immediately removed and tissue specimens were fixed overnight in 4% buffered formaldehyde, tissue processing and paraffin embedded. 3-μm-thick tissue sections were performed for hematoxylin and eosin stain. Tissue was analyzed blindly. Renal tubular injury was scored by estimating the percentage of cortical or outer medullary tubules that showed epithelial necrosis or had luminal necrotic debris and dilation 28, 29, 31. Lesions were graded as follows: 0, none; 1, <5%; 2, 5% to 25%; 3, 25% to 75%; and 4, >75%.

Immunohistochemical (IHC) examinations were performed on 3-μm-thick tissue sections, followed by deparaffinization and rehydration. Antigen was retrieved with a buffer (920P-06, pH 7.3-7.7, Trilogy, Cell Marque, Rocklin, CA, USA) using a microwave oven. After blocking tissue endogenous peroxidase with 3% hydrogen peroxide solution for three minutes, a blocking phosphate buffered saline containing 10% bovine serum albumin was applied for one hour at room temperature. Slides were incubated with an anti-E-cadherin mouse monoclonal antibody (ab47635, Abcam, Cambridge, MA, USA, 1:200 dilution) and an anti-kidney injury molecule-1 (KIM-1) mouse monoclonal antibody (ab76055, Abcam, Cambridge, MA, USA, 1:200 dilution) for 15 minutes at room temperature. Subsequently, the slides were serially rinsed and incubated with biotinylated goat anti-mouse secondary antibodies at room temperature for 30 minutes. After being washed, the slides were incubated in peroxidase-conjugated streptavidin-biotin complex (Dako, Copenhagen, Denmark) for 10 minutes. After applying 3,3ʹ-diaminobenzidine for 1-2 minutes, the sections were counterstained with Mayer hematoxylin, dehydrated with ethanol, and cover slipped for evaluation 29. The mean optical density (i.e., integrated optical density/area) of positive reactions was analyzed with Image-Pro Plus 6.0 software. Total screen area was set as the region of interest for each image. There were ten data points collected from each section, and 8 sections in each group. All scoring was done in a blinded manner.

### Cell culture and drug treatment

NRK-52E cells, which represent immortalized rat tubular epithelial cell line, were purchased from the American Type Culture Collection (Manassas, VA, USA). Briefly, the cells were passed every 3-4 days in 100-mm dishes (Falcon, Bedford, MA, USA) using Dulbecco's modified Eagle's medium-F12 (Sigma-Aldrich, St. Luis, MO, USA) supplemented with 10% fetal bovine serum (Life Technologies Inc., Gaithersburg, MD, USA); insulin-transferrin-sodium selenite media supplement (Sigma-Aldrich, St. Louis, MO, USA); 100 U/mL penicillin; and 100 mg/mL streptomycin (Sigma-Aldrich, St. Louis, MO, USA). For experimental use, these cells were incubated in a humidified atmosphere of 5% CO_2_ and 95% air at 37 °C for 24 hours and subcultured at 70% to 80% confluence.

After being washed extensively with phosphate-buffered saline (PBS), the cells were treated with or without dapagliflozin or LPS for two hours. At the end of treatment, the cells were harvested for molecular analysis. All cellular experiments were repeated three times for semi-quantitative reverse transcription and polymerase chain reaction (RT-PCR).

### Semi-quantitative RT-PCR analysis

The NRK-52E cells were placed on a 6-well culture plate at a density of 5 × 10^5^ cells and incubated overnight. Cells were treated with dapagliflozin with or without LPS (10 μg/mL) in a serum-free medium for 12 and 24 hours. The total RNA from cells was isolated by Trizol reagent (Genepure, Taiwan), according to the manufacturer's instructions. RNA was converted cDNA using random primers by HiSpec Reverse Transcriptase (Yeastern Biotech CO., LTD., Taiwan.). Forty cycles of PCR were performed using 5X Taq PCR MasterMix (Biomate, Taiwan) and the conditions was as follows: 95 °C for 30 seconds, 55-56 °C for 30 seconds and 72 °C for 30 seconds. The specific primers used for PCR amplification are shown as follows Table S1, the specific primers used for PCR amplification included nuclear factor kappa light chain enhancer of activated B cells (NF-κB), chemokine ligand 2 (CCL2), TNF-α, IL-1β, IL-6, AMPK, nuclear factor erythroid 2-related factor 2 (Nrf-2), and heme oxygenase-1 (HO-1). Amplification products were resolved by 2% agarose gel electrophoresis, stained with ethidium bromide, and photographed by Proteinsimple Alphaimager HP and quantified by ImageJ. The mRNA level was calculated by followed: (sample intensity/β-actin intensity of sample)/(control intensity/β-actin intensity of control).

### Reactive oxygen species evaluation

The NRK-52E cells were cultured 4 × 10^5^ cells per 12-well microplate with 5% BS medium for 24 hours. The NRK-52E cells were treated separately with LPS 10 μg/mL, H_2_O_2_ 100 μM, dapagliflozin 4 μg/mL, and LPS + dapagliflozin in serum-free medium for 12 and 24 hours. After sample collection, intracellular reactive oxygen species (ROS) level was determined using 2',7'-dichlorodihydrofluorescein diacetate (DCF-DA) as the fluorescent probe. 4',6-diamidino-2-phenylindole dihydrochloride (DAPI) was used for marking DNA. After incubation with 50-μM DCF-DA for 20 minutes, the cells were treated with 0.5 μg/mL of DAPI for 1 minute. After washing with PBS, DCF-DA and DAPI were determined by fluorescence microscopy measurements 29.

### Statistical analysis

Results were presented as mean ± standard error of the mean (SEM). Data were evaluated by one-way analysis of variance with post-hoc Bonferroni-Dunn for multiple comparisons (SPSS 24.0 for Windows; SPSS, Inc., Chicago, IL, USA). Comparisons between two groups were made by unpaired t-test. P values < 0.05 were considered statistically significant.

## Results

### Dapagliflozin decreased the serum glucose, GOT, and GPT at 48 hours in septic diabetic mice

**Figure 1:**
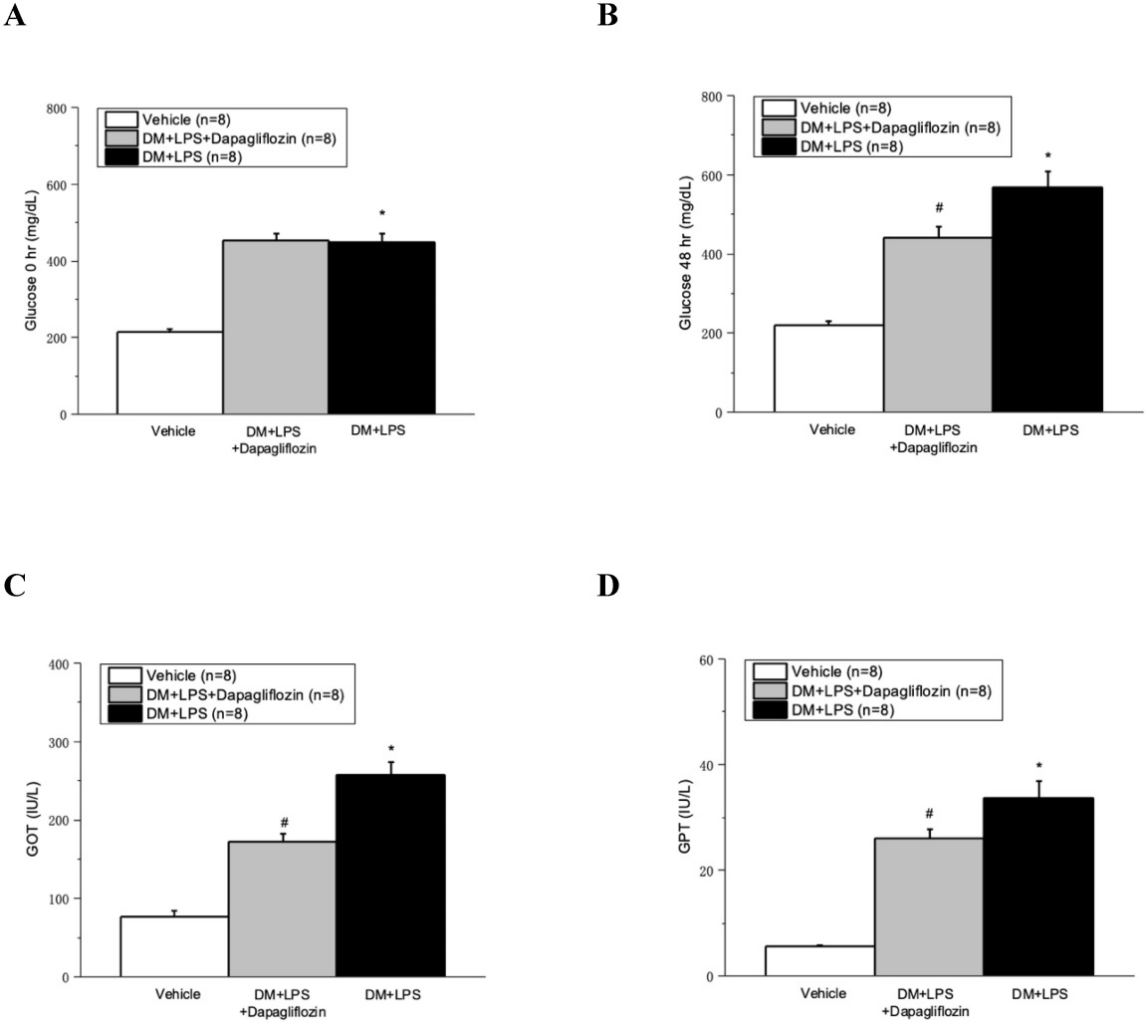
** Dapagliflozin ameliorated glucose change and liver injury in lipopolysaccharide (LPS) induced sepsis in diabetic mice. (A)** Glucose changes at 0 hour after LPS administration. **(B)** Glucose changes at 48 hours after LPS administration. **(C)** Glutamic oxaloacetic transaminase (GOT) changes at 48 hours after LPS administration. **(D)** Glutamic pyruvic transaminase (GPT) changes at 48 hours after LPS administration. * *P* < 0.05 for the DM + LPS group compared with the Vehicle group. # *P* < 0.05 for the DM + LPS + Dapagliflozin group compared with the DM + LPS group.

Compared with the Vehicle group, the DM + LPS and DM + LPS + Dapagliflozin groups demonstrated marked increase of the glucose level in diabetic mice after 48 hours (Fig. 1A and 1B). Compared with the DM + LPS group, the DM + LPS + Dapagliflozin group had significantly decreased blood glucose level at 48 hours after LPS administration (Fig. 1B). The GOT and GPT levels markedly increased after LPS-induced endotoxic shock, compared with those in the vehicle group (Fig. 1C and 1D). Compared with the DM + LPS group, the DM + LPS + Dapagliflozin group had significantly decreased GOT and GPT at 48 hours after LPS administration (Fig. 1C and 1D).

### Mean arterial pressure in septic diabetic mice

**Figure 2:**
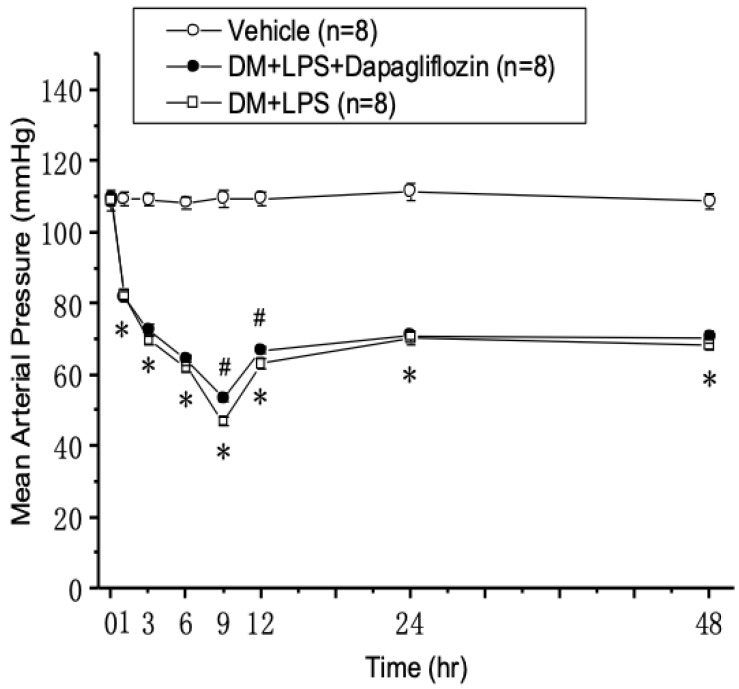
** Changes in mean arterial pressure after endotoxic shock in diabetic mice.** * *P* < 0.05 for the DM + LPS group compared with the Vehicle group. # *P* < 0.05 for the DM + LPS + Dapagliflozin group compared with the DM + LPS group.

After inducing endotoxic shock with LPS for one hour, the mean arterial pressure (MAP) started to markedly decrease in the DM +LPS and DM + LPS + Dapagliflozin groups, compared with that in the vehicle group (Fig. 2). The MAP reached the lowest point at 9 hours and was restored to a relatively steady state at 24 hours in the DM + LPS and DM + LPS + Dapagliflozin groups. Compared with the DM + LPS group, the DM + LPS + Dapagliflozin group had significantly increased MAP at 9 and 12 hours after endotoxic shock.

### Dapagliflozin ameliorated LPS-induced AKI in diabetic mice

**Figure 3:**
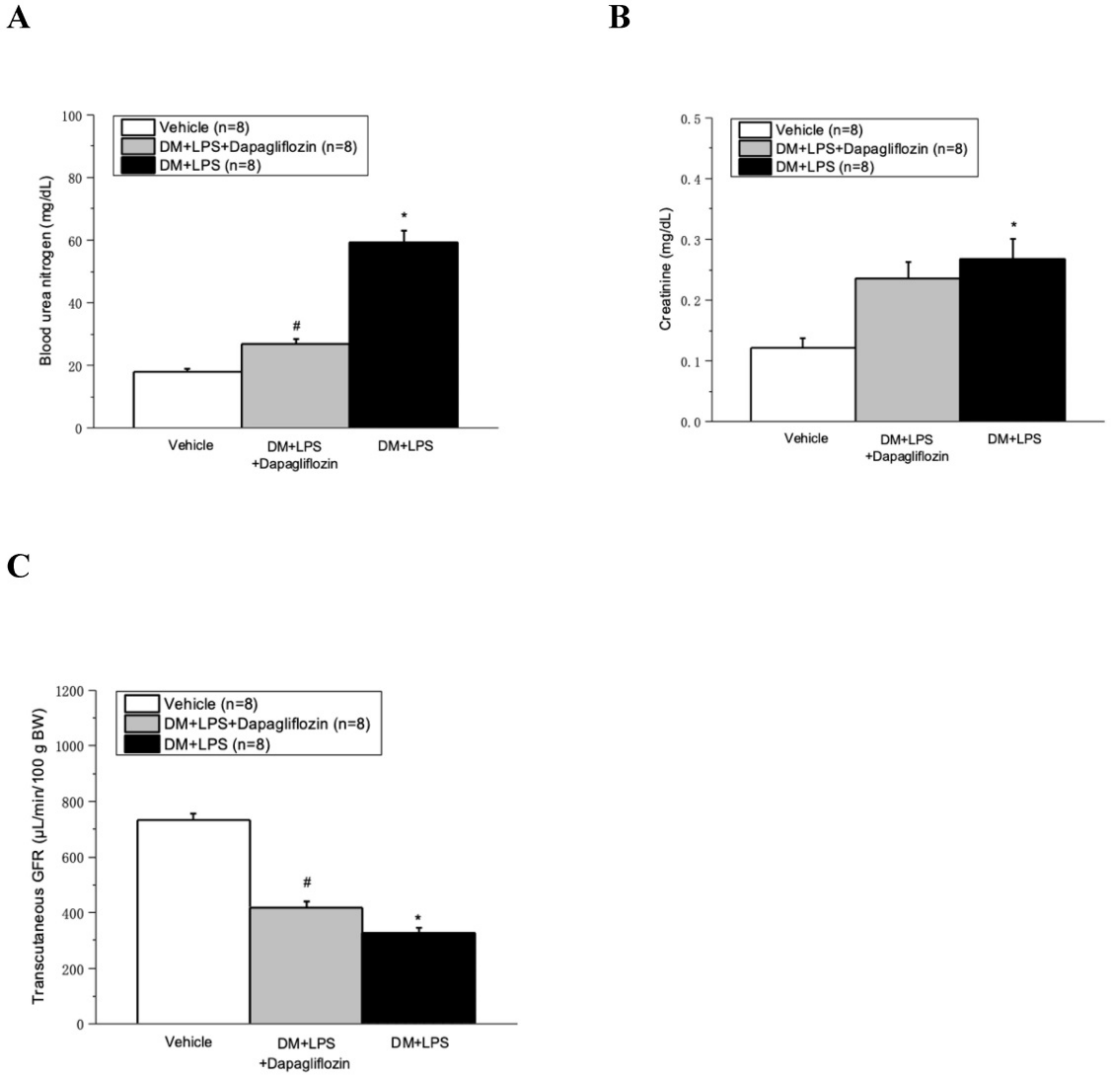
** Dapagliflozin ameliorated kidney injury in lipopolysaccharide (LPS) induced sepsis in diabetic mice. (A)** Blood urea nitrogen, **(B)** Creatinine, and **(C)** FITC-sinistrin transcutaneous glomerular filtration rate (GFR) at 48 hours after LPS administration. * *P* < 0.05 for the DM + LPS group compared with the Vehicle group. # *P* < 0.05 for the DM + LPS + Dapagliflozin group compared with the DM + LPS group.

The serum BUN and creatinine significantly increased, whereas the transcutaneous GFR significantly decreased in the DM + LPS and DM + LPS + Dapagliflozin groups, compared with those in the vehicle group (Fig. 3A-3C). Compared with the DM + LPS group, the DM + LPS + Dapagliflozin group had significantly decreased BUN and increased transcutaneous GFR at 48 hours after LPS administration (Fig. 3B and 3C).

**Figure 4:**
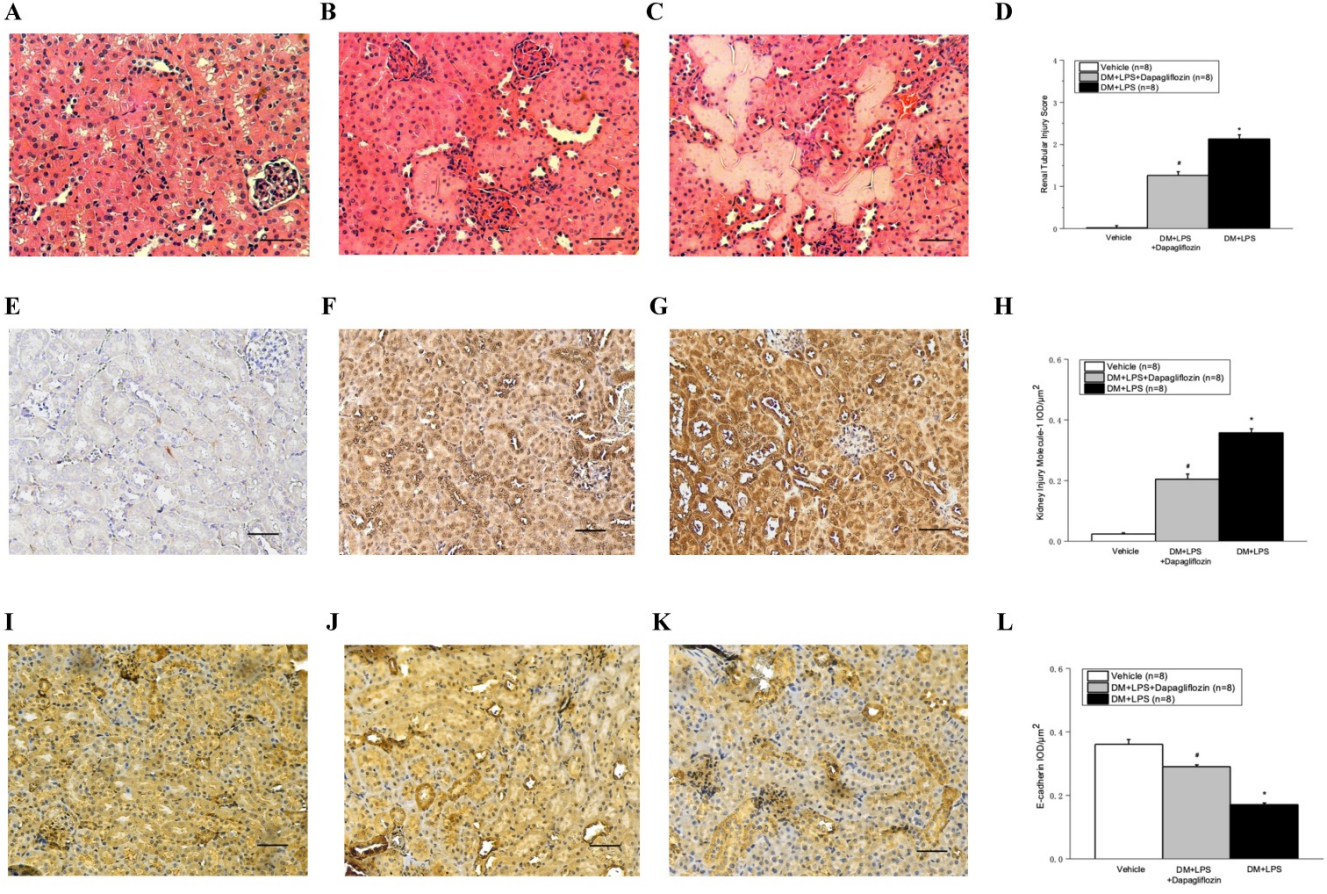
** Dapagliflozin ameliorated histopathologic changes in kidneys in lipopolysaccharide (LPS) induced sepsis in diabetic mice. (A-C)** Hematoxylin and eosin stain (magnification 200x). **(E-G)** Kidney injury molecule-1 (KIM-1) immunohistochemical stain. **(I-K)** E-cadherin immunohistochemical stain. **(A, E, I)** Vehicle group, **(B, F, J)** DM + LPS+ Dapagliflozin group, and **(C, G, K)** DM + LPS group. **(D)** Renal tubular injury score. **(E)** Semi-quantitative evaluation of KIM-1 expression represented as IOD/µm^2^. **(L)** Semi-quantitative evaluation of E-cadherin expression represented as IOD/µm^2^. IOD: integrated optical density. * *P* < 0.05 for the DM + LPS group compared with the Vehicle group. # *P* < 0.05 for the DM + LPS + Dapagliflozin group compared with the DM + LPS group. Scale bar represents as 50 µm in length.

Histopathologic analysis of kidney tissues after LPS-induced endotoxic shock showed tubular swelling, tubular necrosis, and hemorrhage (Fig. 4C). In addition, LPS-induced endotoxic shock increased the expression of KIM-1 and decreased the expression of E-cadherin (Fig. 4G and 4K). Compared with the DM + LPS group, the DM + LPS + Dapagliflozin group had significantly decreased renal tubular injury score and KIM-1 IHC stain and increased E-cadherin stain (Fig. 4D, 4H, and 4L).

### Dapagliflozin ameliorated LPS-induced inflammatory cytokine reactions in diabetic mice

**Figure 5:**
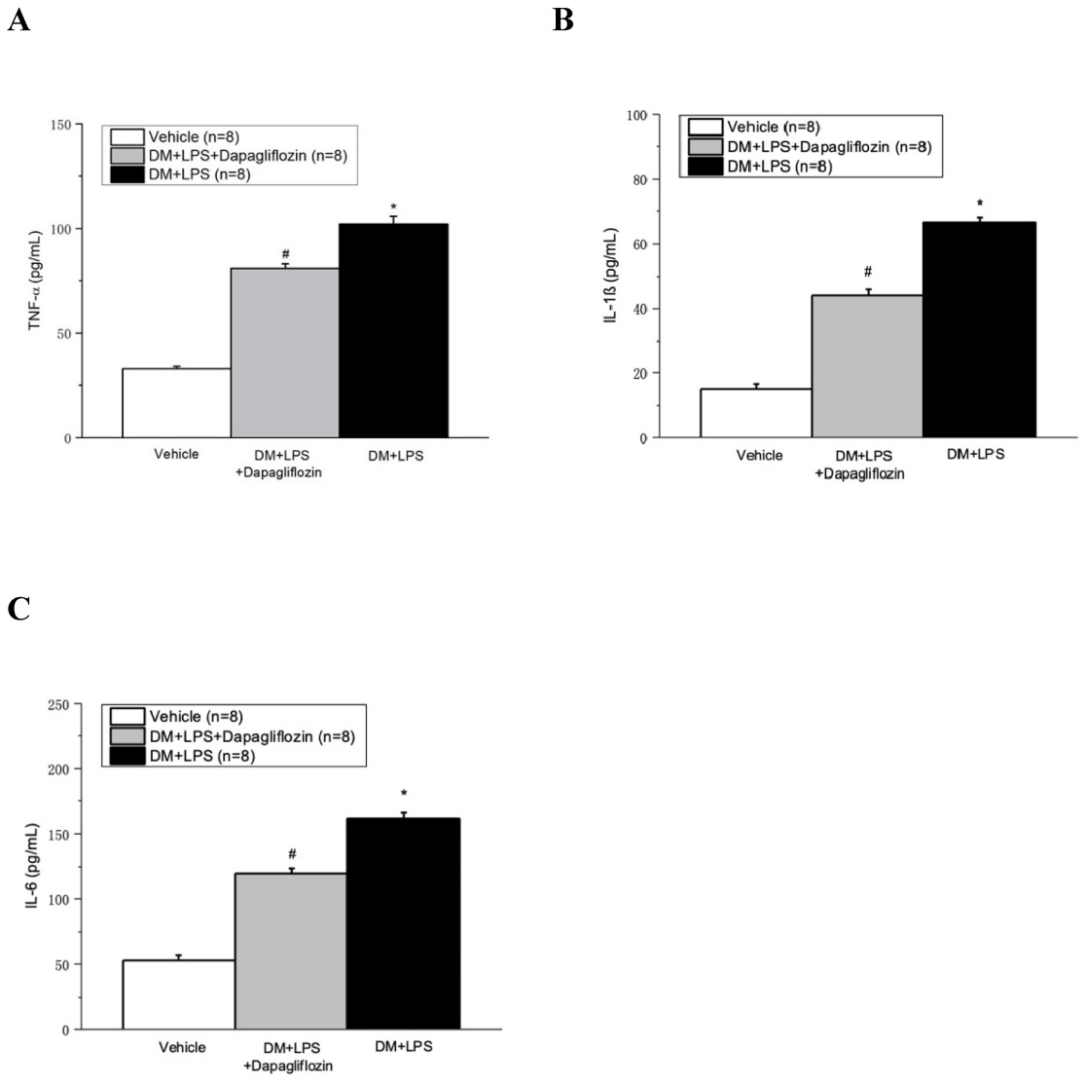
** Dapagliflozin decreased pro-inflammatory cytokines in lipopolysaccharide (LPS) induced sepsis in diabetic mice. (A)** TNF-α, **(B)** IL-1β, and **(C)** IL-6 at 48 hours after LPS administration. * *P* < 0.05 for the DM + LPS group compared with the Vehicle group. # *P* < 0.05 for the DM + LPS + Dapagliflozin group compared with the DM + LPS group.

As shown in Fig. 5, compared with the vehicle group, the DM + LPS group had markedly elevated serum levels of TNF-α, IL-1β, and IL-6. Compared with the DM + LPS group, the DM + LPS + Dapagliflozin group had significantly decreased levels of TNF-α, IL-1β, and IL-6.

### Dapagliflozin decreased the mRNA expressions of inflammatory cytokines and activated the mRNA expression of the AMPK pathway in LPS-induced endotoxic shock

**Figure 6:**
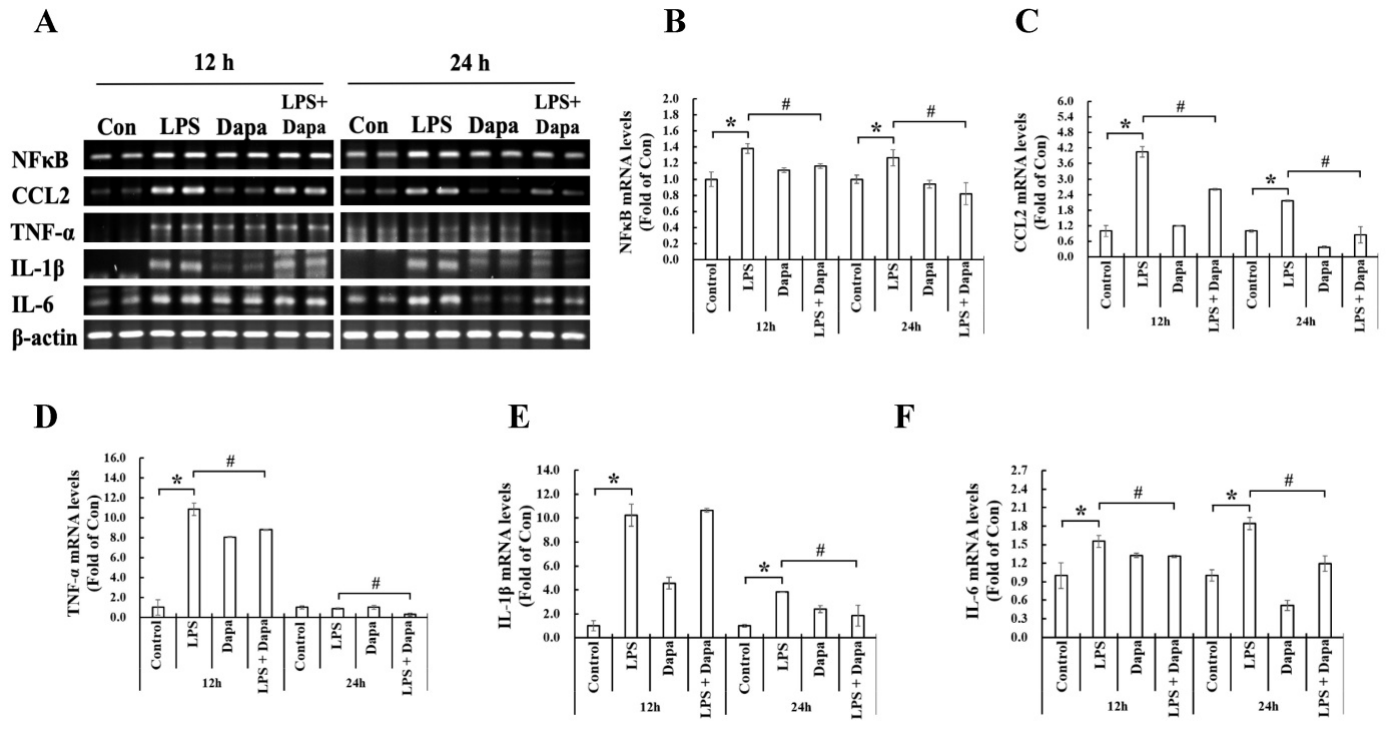
** Dapagliflozin can inhibit mRNA expression of inflammatory mediators.** Semi-quantitative reverse transcription and polymerase chain reaction (RT-PCR) of NRK-52E cells after 12 or 24 h incubation with/without 10 µg/mL lipopolysaccharide (LPS) or 4 µg/mL dapagliflozin (Dapa). **(A)** RT-PCR analyses of NF-κB, CCL2, TNF-α, IL-1β, and IL-6. The semi-quantification results of RT-PCR for **(B)** NF-kB, **(C)** CCL2, **(D)** TNF-α, **(E)** IL-1β and **(F)** IL-6. * *P* < 0.05 for the LPS group compared with the Vehicle group. # *P* < 0.05 for the LPS + Dapa group compared with the LPS group. Each n = 4.

Compared with the control NRK-52E cells, the NRK-52E cells treated with LPS for 12 and 24 hours showed significantly elevated mRNA of the transcription factor (NF-κB) and inflammatory cytokines (CCL2, TNF-α, IL-1β, and IL-6). Compared with the NRK-52E cells treated with LPS, those treated with LPS + Dapagliflozin for 12 and 24 hours had significantly decreased inflammatory cytokines. These findings are shown in Fig. 6.

**Figure 7:**
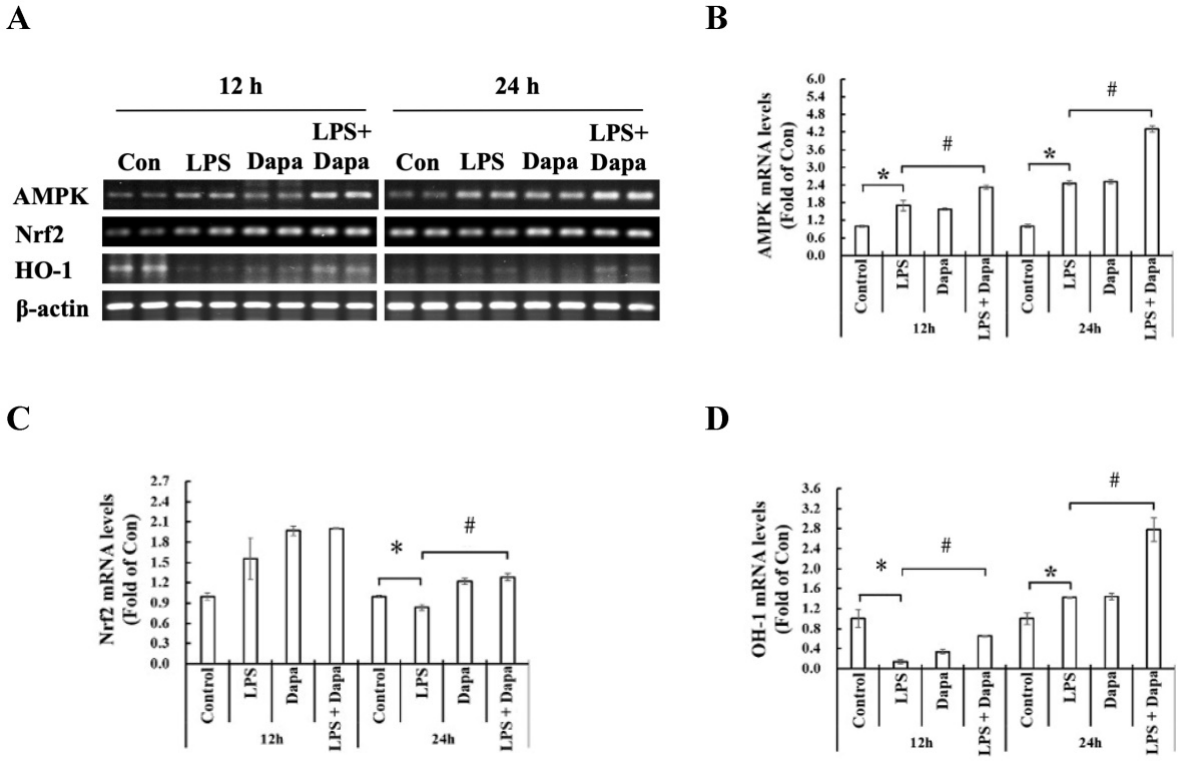
** Dapagliflozin can activate mRNA expression of AMPK pathway.** Semi- quantitative reverse transcription and polymerase chain reaction (RT-PCR) of NRK-52E cells after 12 or 24 h incubation with/without 10 µg/mL lipopolysaccharide (LPS) or 4 µg/mL dapagliflozin (Dapa). **(A)** Semi-quantitative RT-PCR analyses of AMP-activated protein kinase (AMPK), nuclear factor erythroid 2-related factor 2 (Nrf-2), and heme oxygenase-1 (HO-1). The semi-quantification results of RT-PCR for **(B)** AMPK, **(C)** Nrf-2, and **(D)** HO-1. * *P* < 0.05 for the LPS group compared with the Vehicle group. # *P* < 0.05 for the LPS + Dapa group compared with the LPS group. Each n = 4.

Furthermore, compared with the control NRK-52E cells, the NRK-52E cells treated with LPS for 12 and 24 hours showed increased expressions of the AMPK pathway mRNAs (i.e., AMP kinase, Nrf-2, and HO-1). In addition, the mRNA expressions of the AMPK pathway were more activated in the NRK-52E cells treated with LPS + Dapagliflozin than in the NRK-52E cells treated with LPS. These findings are shown in Fig. 7.

### Dapagliflozin decreased ROS in LPS-induced endotoxic shock

**Figure 8:**
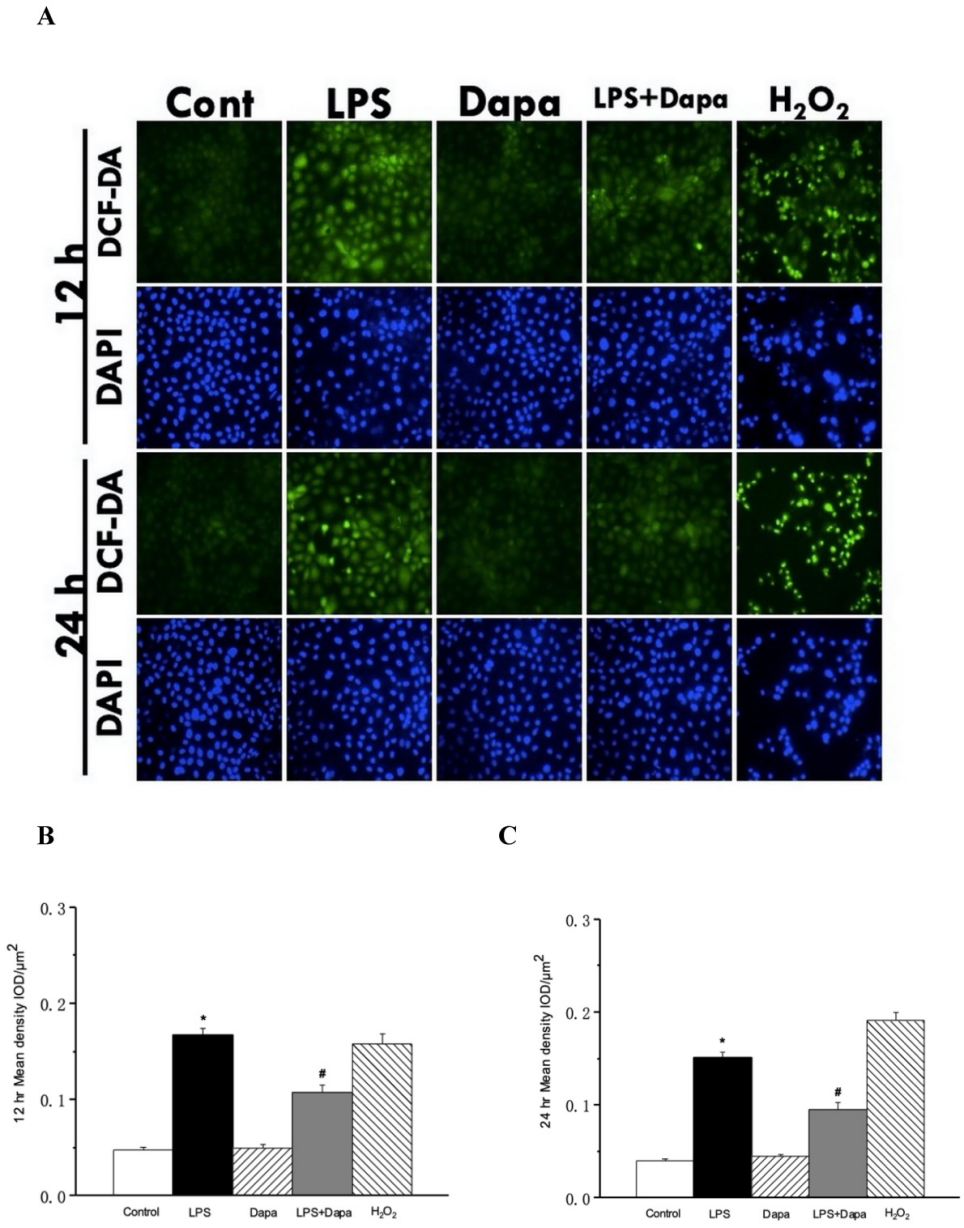
** Dapagliflozin can reduce reactive oxygen species (ROS) in sepsis.** ROS assay of NRK-52E cells after 12 or 24 h incubation with/without 10 µg/mL lipopolysaccharide (LPS) or 4 µg/mL dapagliflozin (Dapa), using 2',7'-dichlorodihydrofluorescein diacetate (DCF-DA) as a fluorescent probe and 4',6-diamidino-2-phenylindole dihydrochloride (DAPI) to stain the nuclear acid. **(A)** 4 µg/mL Dapa attenuated ROS induced by 10 µg/mL LPS in NRK-52E cells. Semi-quantitative evaluation of ROS expression represented as IOD/µm^2^ at **(B)** 12 h **(C)** 24 h. IOD: integrated optical density. * P < 0.05 for the LPS group compared with the Vehicle group. # P < 0.05 for the LPS + Dapa group compared with the LPS group. Each n = 4.

As shown in Fig. 8, LPS increased the ROS in the NRK-52E cells. After treatment with dapagliflozin, suppression of the DCF-DA fluorescence intensity was augmented by 12 and 24 hours of LPS-induced production of ROS in the NRK-52E cells.

## Discussion

Our study showed that dapagliflozin can attenuate LPS-induced endotoxic shock associated with AKI in streptozotocin-induced diabetic mice. In addition, dapagliflozin reduced the inflammatory cytokines and ROS by activating the AMPK pathway in the NRK-52E cells with LPS-induced sepsis. Dapagliflozin lowers the blood sugar level by decreasing glucose reabsorption in the proximal renal tubule. In addition to its effects on lowering blood sugar, dapagliflozin was shown by clinical trials and animal studies to inhibit the inflammatory response 32-35 and reduce ROS production 34, 36 in diabetes.

DM is considered as a chronic inflammation status and usually causes target organ damage by prolonged increase of oxidative stress and ROS 37. Similarly, sepsis is characterized by inflammation and increased ROS production 38. The effect of SGLT2 inhibitors in sepsis is unknown. In our study, we demonstrated the protective effect of dapagliflozin on LPS-induced endotoxic shock with AKI in streptozotocin-induced diabetic mice, based on the increased FITC-sinistrin GFR, decreased renal tubular injury score and KIM-1 IHC staining, and increased E-cadherin staining. Moreover, dapagliflozin was found to reduce the production of the inflammatory cytokines TNF-α, IL-1β, and IL-6. These results indicated that the beneficial effects of dapagliflozin were mediated by amelioration of the AKI in LPS-induced endotoxic shock in diabetic mice through inhibition of the inflammatory process.

In addition to its effects on maintaining cellular energy balance 39, the AMPK pathway can negatively regulate immune cells and suppress sepsis development 9. Activation of the AMPK pathway is a physiologic anti-inflammatory process. Several *in vitro* studies showed that dapagliflozin can activate the AMPK pathway thereby inhibiting sepsis-associated severe inflammation in renal epithelial cells 25 or podocyte 24 or cardiofibroblasts 40, not directly through immune cells. In our study of NRK-52E cells treated with LPS, dapagliflozin inhibited the production of inflammatory cytokines and ROS and activated AMPK, HO-1, and Nrf-2. These results suggested that dapagliflozin can rescue LPS-induced damage by activating the AMPK pathway in renal epithelial cells.

There is a limitation in our study. We do not perform mRNA or protein analysis in animal kidney tissue, to provide a confirmation that the attenuation of inflammatory cytokines expression *in vivo*. However, we performed the cell culture study shows mitigating inflammatory effect of SGLT2 inhibitor on renal tubular cells, indirectly indicating its possible improvement *in vivo*.

In conclusion, this study showed that dapagliflozin can attenuate LPS-induced endotoxic shock with AKI in streptozotocin-induced diabetic mice by decreasing inflammatory cytokine production. Activation of the AMPK pathway, suppression of pro-inflammatory cytokines, and attenuation of ROS production by dapagliflozin treatment may play an important role in the protection from LPS-induced renal damage.

## Supplementary Material

Supplementary table.

## Abbreviations

AKI: Acute kidney injury
AMPK: Adenosine monophosphate-activated protein kinase
BUN: Blood urea nitrogen
CCL2: Chemokine ligand 2
DAMP: Damage associated with molecular pattern
DM: Diabetes mellitus
FITC: Fluorescein isothiocyanate
GFR: Glomerular filtration rate
GOT: Glutamate oxaloacetate transaminase
GPT: Glutamic pyruvic transaminase
HO-1: heme oxygenase-1
IL-6: interleukin-6
KIM-1: Kidney injury molecule-1
LPS: Lipopolysaccharide
MAP: Mean arterial pressure
NF-κB: Nuclear factor kappa light chain enhancer of activated B cells
Nrf-2: Nuclear factor erythroid 2-related factor 2
PAMP: Pathogen associated molecular pattern
PBS: Phosphate-buffered saline
PRR: Pathogen recognition receptors
ROS: Reactive oxygen species
RT-PCR: reverse transcription and polymerase chain reaction
SEM: standard error of the mean
SGLT2: Sodium-glucose cotransporter 2
TNF-α: tumor necrosis factor-α
