# Supplementary material for: Dapagliflozin Ameliorates Lipopolysaccharide Related Acute Kidney Injury in Mice with Streptozotocin-induced Diabetes Mellitus
Source: Int J Med Sci. 2022 Apr 4;19(4):729–39. doi: 10.7150/ijms.69031 (PMC9108401; doi:10.7150/ijms.69031)
Supplement: Supplementary file 1 — Supplementary table. [file ijmsv19p0729s1.pdf]

Supplementary file

Table S1. Primer sequences used for semi-quantitative reverse transcription and polymerase chain reaction (RT-PCR) analysis.

	Forward Primer (5'-3')	Reverse Primer (5'-3')
NF-κB	AACAACACAGACCCAGGAGT	CTGTCACCAGGCGAGTTATAG
CCL2	GTCACCAAGCTCAAGAGAGAGA	GAGTGGATGCATTAGCTTCAGA
TNF-α	CGAGTCTGGGCAGGTCTACTTT	AGAGGTTGAGGGTGTCTGAAGG
IL-1β	CCCTGAACTCAACTGTGAAATAGCA	CCCAAGTCAAGGGCTTGGAA
IL-6	GAGTTGTGCAATGGCAATTC	ACTCCAGAAGACCAGAGCAG
AMPK	CGGCAAAGTGAAGGTTGGCAAA	CAAATAGCTCTCCTCCTGAGAC
Nrf2	TCTGACTCCGGCATTTCACT	GGCACTGTCTAGCTCTTCCA
OH-1	CACGCATATACCCGCTACCT	CCAGAGTGTTCATTCGAGA
β-actin	AAGTCCCTCACCCTCCCAAAAG	AAGCAATGCTGTCACCTTCCC
